# From Leiomyoma to Smooth Muscle Tumor of Uncertain Malignant Potential: A Rare Recurrent Right Retroperitoneal Mass in a Young Male

**DOI:** 10.7759/cureus.115494

**Published:** 2026-08-31

**Authors:** Yeshavanth Preetham, Yuvaraja Thyavihally, Manit Gundavda, Bijal Kulkarni, Akash Shah

**Affiliations:** 1 Uro-Oncology, Kokilaben Dhirubhai Ambani Hospital, Mumbai, IND; 2 Orthopaedic Oncology, Kokilaben Dhirubhai Ambani Hospital, Mumbai, IND; 3 Pathology, Kokilaben Dhirubhai Ambani Hospital, Mumbai, IND

**Keywords:** leiomyoma, male patient, retroperitoneal neoplasm, retroperitoneal stump, smooth muscle tumor, tumor recurrence

## Abstract

Smooth muscle tumors of uncertain malignant potential (STUMP) are rare mesenchymal neoplasms that exhibit histopathological features intermediate between benign leiomyoma and leiomyosarcoma. STUMP is overwhelmingly a disease of the uterus in perimenopausal and postmenopausal women; extra-uterine STUMPs are exceedingly uncommon, and involvement of the retroperitoneum in a male patient is exceptionally rare, with only a handful of cases described in the literature to date. We report a unique case of a young male who initially underwent excision of a retroperitoneal leiomyoma and subsequently developed a recurrent retroperitoneal STUMP after a six-year disease-free interval. A 25-year-old male initially presented with a progressively enlarging right flank mass and underwent complete excision. Histopathology revealed a benign retroperitoneal leiomyoma. The patient was lost to follow-up and re-presented six years later with a recurrent right retroperitoneal mass. Imaging demonstrated a heterogeneously enhancing, dumbbell-shaped lesion at the previous operative site with extension toward the deep subcutaneous plane and proximity to the right iliac crest. Wide local excision with periosteal margin resection and abdominal wall reconstruction using polypropylene mesh was performed. Histopathology demonstrated a cellular spindle cell neoplasm with elevated mitotic activity (18-20 mitoses/10 high-power fields) but without significant nuclear atypia, atypical mitoses, or coagulative tumor necrosis. Immunohistochemistry was positive for smooth muscle actin (SMA) and desmin, with aberrant MyoD1 expression, confirming smooth muscle differentiation. The lesion was diagnosed as a cellular, mitotically active STUMP. Follow-up imaging seven months postoperatively demonstrated no evidence of recurrence. This case highlights the rare occurrence of recurrent retroperitoneal STUMP in a young male - a sex in which this diagnosis is only anecdotally reported - and suggests possible biological progression along the leiomyoma-STUMP spectrum.

## Introduction

Smooth muscle tumors of uncertain malignant potential (STUMP) represent a rare and diagnostically challenging group of smooth muscle neoplasms that cannot be definitively classified as benign leiomyoma or malignant leiomyosarcoma. The diagnosis rests on the Stanford criteria of Bell et al., which weigh three histological parameters - cytological atypia, mitotic activity, and coagulative tumor necrosis - to place a tumor along the leiomyoma-STUMP-leiomyosarcoma spectrum [1,2].

STUMP is, in practice, almost entirely a disease of the uterus. Contemporary systematic reviews describe it as a tumor of perimenopausal and postmenopausal women, typically diagnosed between 45 and 55 years of age [3]. Extra-uterine STUMPs - arising in the retroperitoneum, soft tissue, bladder, or other sites without a uterine primary - are markedly rarer still, and reports of STUMP in male patients are exceptionally uncommon, largely confined to isolated case reports [4-8]. This sex disparity is not incidental: because the diagnostic criteria, expected biological behavior, and follow-up algorithms for STUMP were all derived from uterine (i.e., exclusively female) cohorts, a STUMP arising outside the female genital tract - and especially in a male patient - falls outside the population in which this diagnosis has been validated, making both its interpretation and its management genuinely uncertain.

The biological behavior of STUMP remains unpredictable, with reported risks of local recurrence and occasional malignant transformation even in the better-studied uterine setting. Because of the rarity of extra-uterine and male disease, management strategies for these patients are almost entirely extrapolated from experience with uterine smooth muscle tumors. We present a rare case of recurrent retroperitoneal STUMP in a young male - one of only a small number of male STUMP cases reported to date, and, to our knowledge, the only one to have arisen at the exact site of a previously excised, histologically confirmed benign leiomyoma - developing six years after the index surgery. This report is prepared in accordance with the CARE (CAse REport) guidelines for case report writing.

## Case presentation

A 25-year-old male initially presented with complaints of a progressively enlarging right flank swelling associated with pain for three months. He also reported loss of appetite and weight loss. Physical examination revealed a firm right lumbar mass with restricted mobility.

Contrast-enhanced computed tomography demonstrated a large, heterogeneously enhancing soft tissue mass measuring approximately 10.5 × 6.7 × 5.5 cm in the right iliac fossa and lumbar region, extending into the adjacent deep subcutaneous plane and exerting mass effect on the psoas muscle (Figure 1).

**Figure 1:**
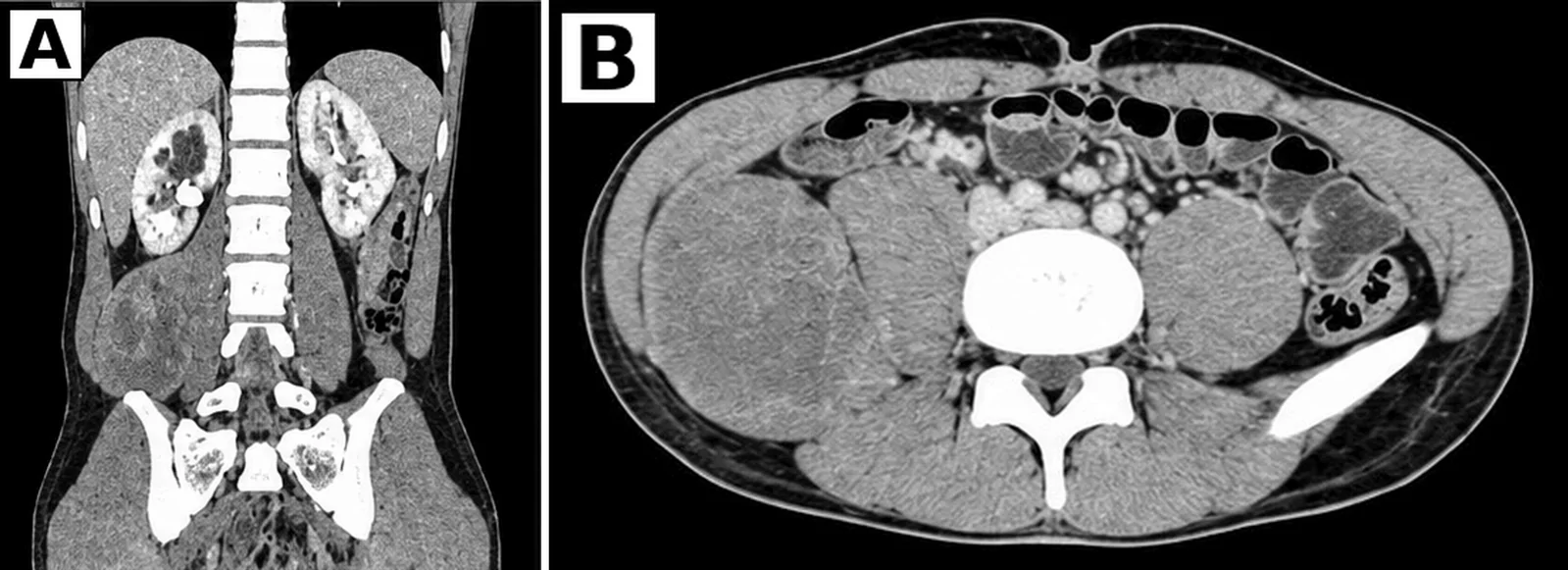
Contrast-enhanced CT abdomen demonstrating the primary right retroperitoneal mass prior to first excision. (A) Coronal reconstruction; (B) Axial image showing the large right-sided retroperitoneal mass (10.5 × 6.7 × 5.5 cm).

The patient subsequently underwent open excision of the retroperitoneal mass. Intraoperatively, a large lobulated mass was identified in the right retroperitoneum and excised completely. Histopathological examination revealed a benign retroperitoneal leiomyoma with negative margins (Figure 2).

**Figure 2:**
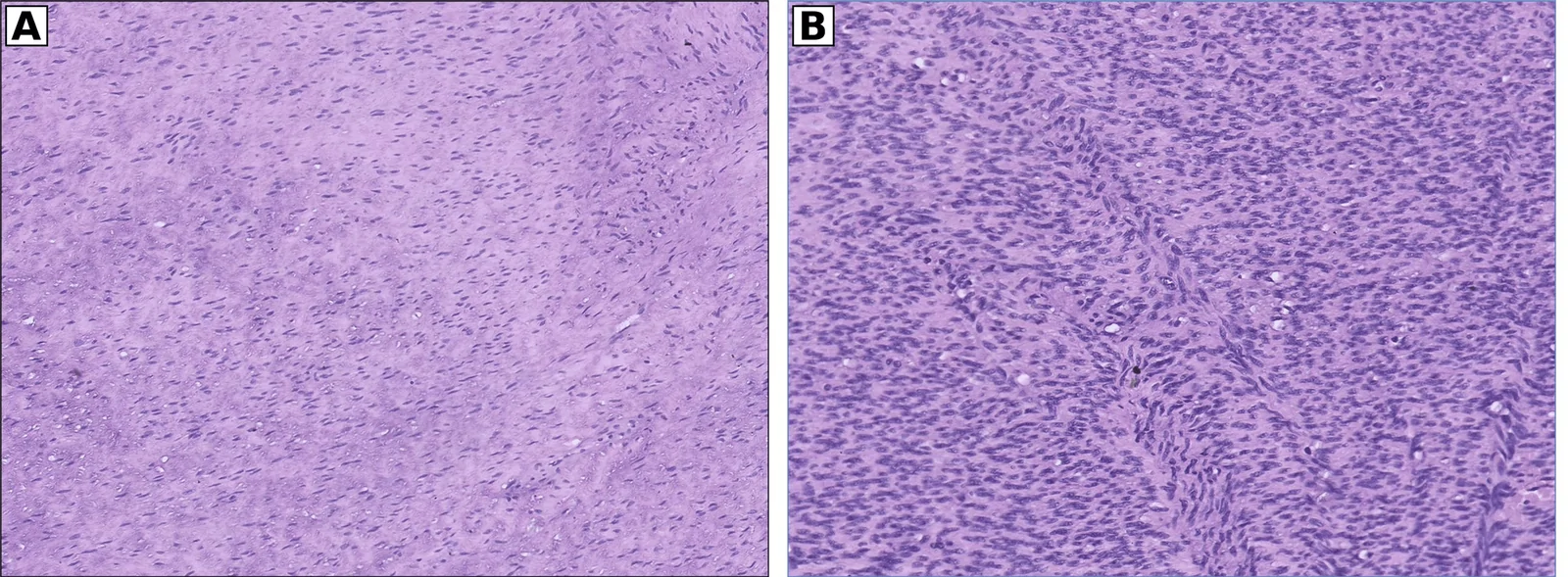
Histopathological photomicrograph (H&E) of the original excised retroperitoneal leiomyoma. (A) Spindle cell tumor with low cellularity (H&E, original magnification ×10); (B) Focal, more cellular area, without increased mitotic activity, nuclear atypia, or necrosis (H&E, original magnification ×100).

The patient remained asymptomatic but was subsequently lost to follow-up. Six years after the initial surgery, he noted a palpable swelling at the operative site and presented for evaluation, at which point recurrence was confirmed on ultrasonography. Ultrasonography demonstrated a well-defined retroperitoneal solid lesion measuring 4.5 × 2.7 × 4.0 cm. Contrast-enhanced CT of the abdomen revealed a heterogeneously enhancing, lobulated, dumbbell-shaped soft tissue mass measuring 5.4 × 4.7 × 3.7 cm in the right retroperitoneum posterior to the cecum, at the site of the previous surgery. The lesion extended toward the adjacent deep subcutaneous plane and was noted to be flush with the right iliac crest with subtle cortical blunting (Figure 3).

**Figure 3:**
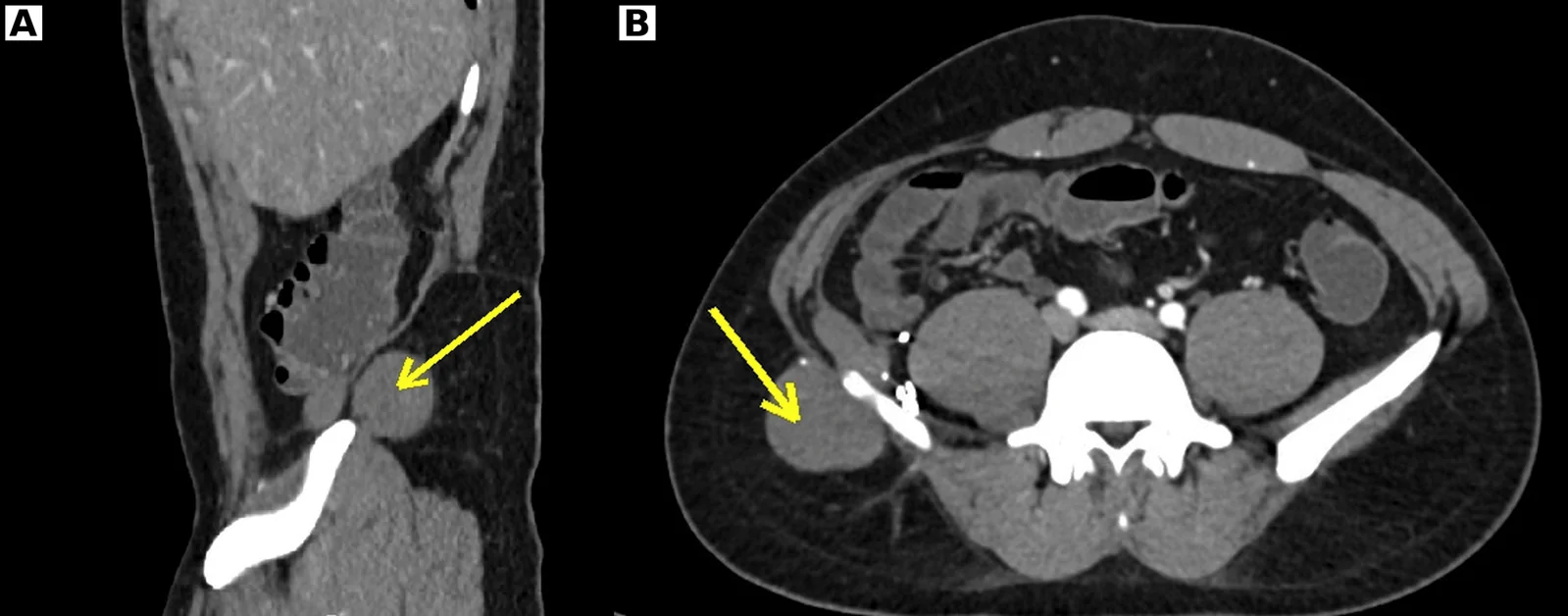
Axial and sagittal CT images of the recurrent retroperitoneal mass. (A) Sagittal CT image demonstrating the recurrent retroperitoneal lesion, posterior to the cecum at the prior operative site (arrow). (B) Axial CT image demonstrating extension of the mass outside the abdomen into the subcutaneous plane, flush with the right iliac crest (arrow).

Given the recurrence and concern regarding involvement of the iliac crest, a multidisciplinary surgical approach involving surgical oncology, orthopedic surgery, and plastic surgery was planned.

The patient subsequently underwent wide local excision of the recurrent retroperitoneal mass with periosteal resection of the iliac crest margin. Following tumor excision, abdominal wall reconstruction was performed using polypropylene mesh (Figure 4).

**Figure 4:**
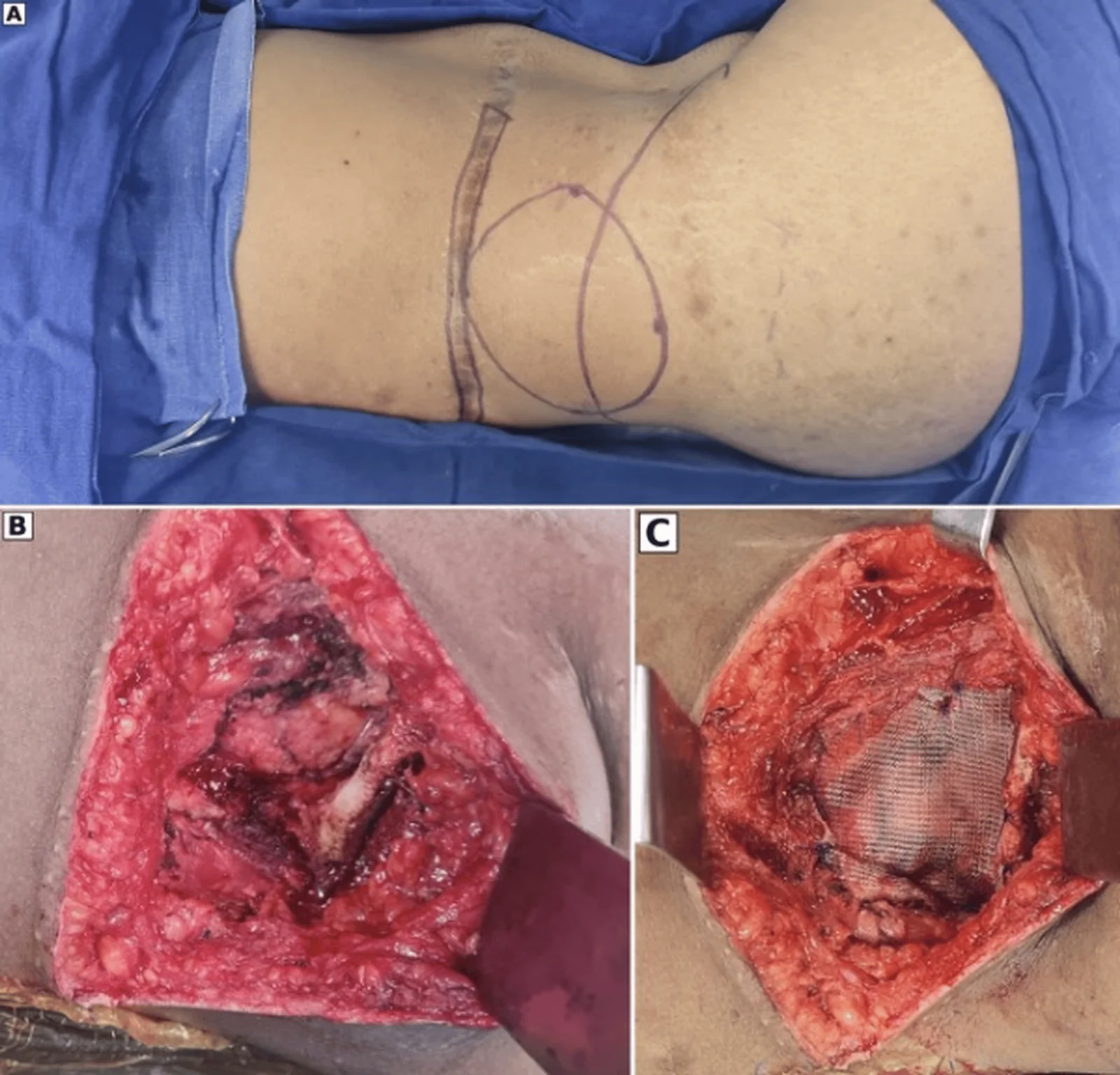
Intraoperative photographs of wide local excision and abdominal wall reconstruction. (A) Preoperative marking of the recurrent mass and previous surgical scar prior to wide local excision. (B) Intraoperative view of the excision bed following wide local excision and periosteal resection, showing the exposed right iliac crest margin. (C) Abdominal wall reconstruction with polypropylene mesh secured to the iliac crest and surrounding soft tissue.

Gross examination demonstrated a well-circumscribed retroperitoneal specimen measuring 8 × 6.7 × 6.1 cm; on cut section, a circumscribed, thinly encapsulated tumor nodule measuring 5 × 4.5 × 4 cm was identified within the surrounding fat (Figure 5).

**Figure 5:**
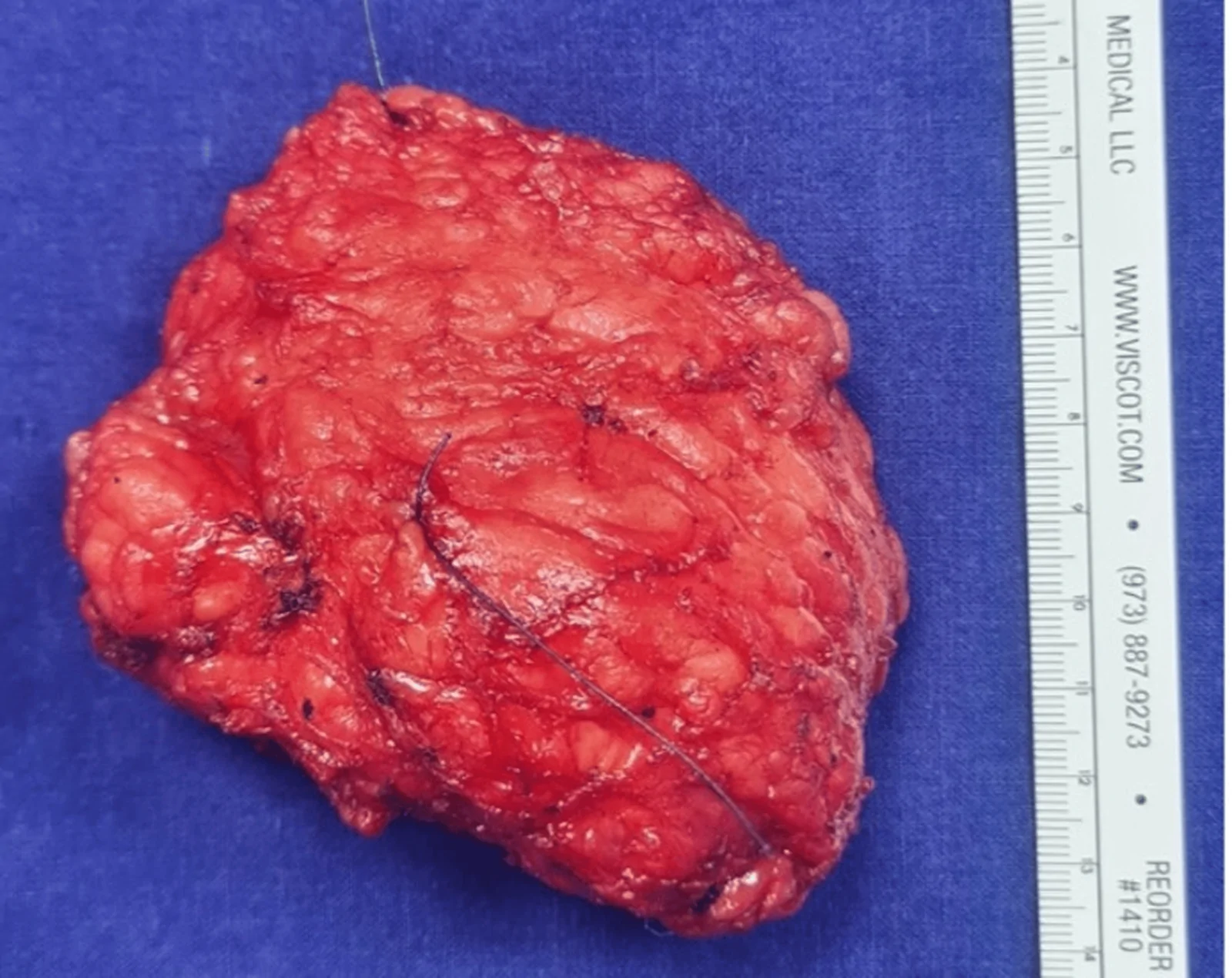
Gross specimen of the recurrent retroperitoneal tumor. External surface of the resected mass, a well-circumscribed, lobulated specimen measuring 8 cm in maximum dimension (ruler shown in cm for scale).

Microscopic examination revealed a cellular spindle cell neoplasm composed of fascicles of smooth muscle cells. Mitotic activity was markedly increased, at approximately 18-20 mitoses per 10 high-power fields. However, there was no significant nuclear atypia, atypical mitotic activity, or coagulative tumor necrosis. Surgical margins were free of tumor (Figure 6).

**Figure 6:**
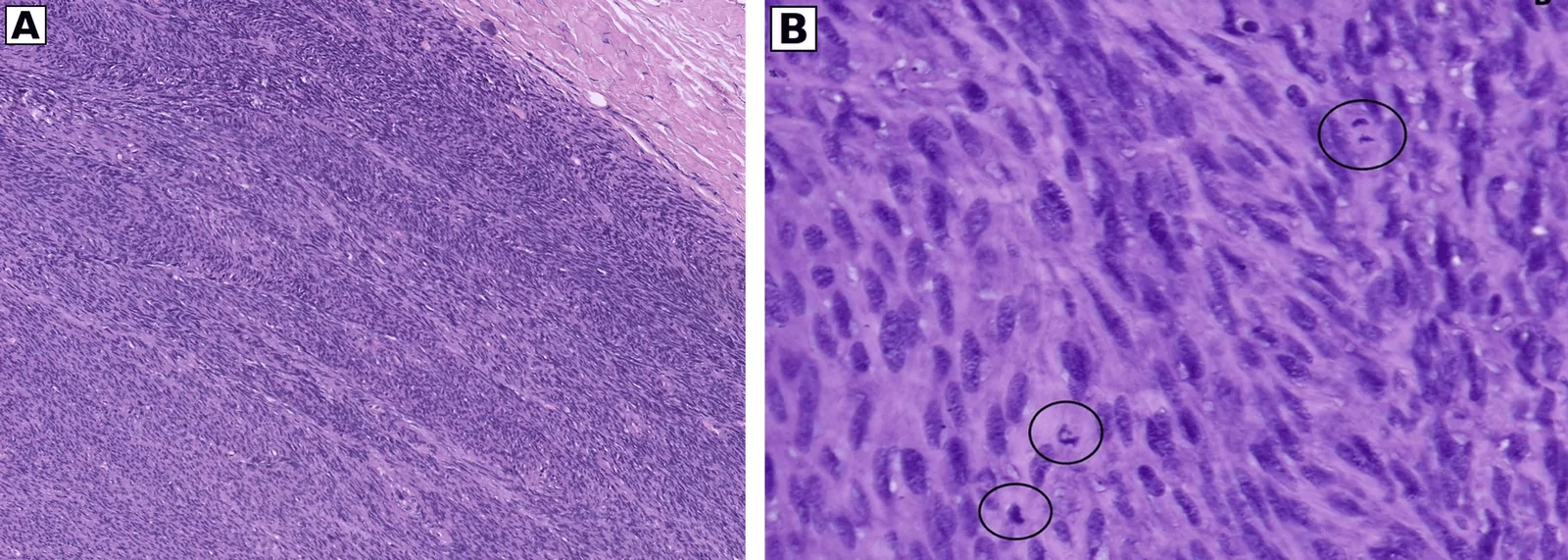
Histopathological examination (H&E) of the recurrent tumor. (A) Cellular spindle cell neoplasm arranged in fascicles, with markedly increased cellularity compared with the original specimen (H&E, original magnification ×10); (B) High-power field with circled mitotic figures, corresponding to the elevated mitotic index (18–20/10 HPF), the key diagnostic finding distinguishing this lesion from the original leiomyoma, without accompanying nuclear atypia or coagulative necrosis (H&E, original magnification ×100).

Immunohistochemistry demonstrated diffuse positivity for smooth muscle actin (SMA) and desmin, with aberrant MyoD1 expression. Tumor cells were negative for myogenin, h-caldesmon, DOG1, CD34, STAT6, SOX10, S100, c-kit, and MDM2, a panel performed to confirm smooth muscle lineage and exclude morphological mimics.

Review of the original histological slides (Figure 2) confirmed a benign retroperitoneal leiomyoma. Compared with the original tumor, the recurrent lesion demonstrated increased cellularity and substantially higher mitotic activity. Based on the absence of significant atypia and tumor necrosis despite elevated mitotic activity, a diagnosis of cellular, mitotically active smooth muscle tumor of uncertain malignant potential (STUMP) was rendered.

The postoperative course was uneventful. Follow-up CT imaging performed seven months after surgery, the most recent assessment available at the time of this report, demonstrated no evidence of local or regional recurrence (Figure 7).

**Figure 7:**
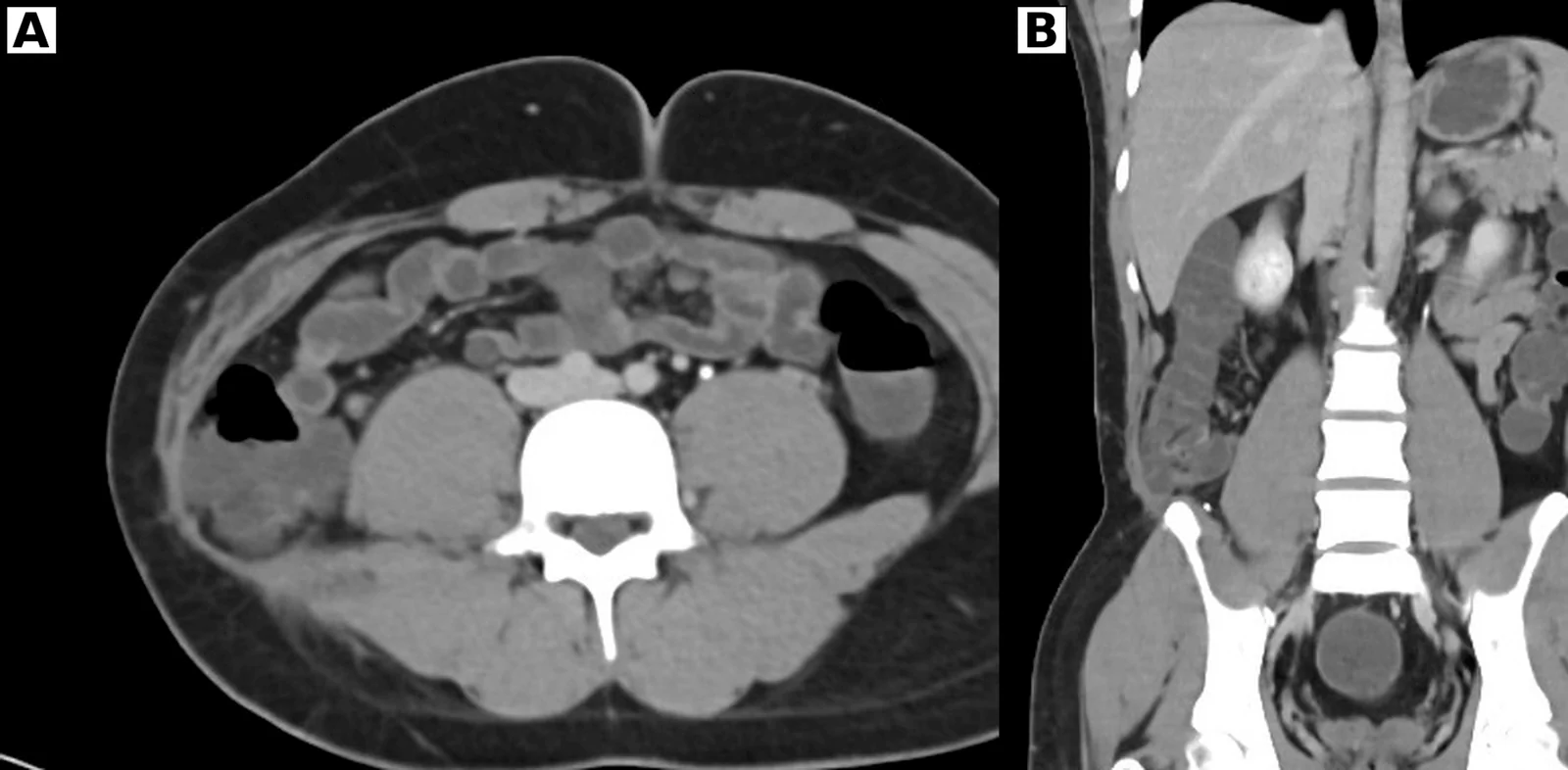
Follow-up CT at seven months showing no evidence of recurrence. (A) Axial and (B) coronal contrast-enhanced CT images at the right retroperitoneal operative site, showing post-excision changes without evidence of local or regional recurrence.

## Discussion

STUMP was first described in the uterine literature by Kempson in 1973 and formalized by Bell, Kempson, and Hendrickson in their 1994 analysis of 213 problematic uterine smooth muscle tumors, which established the mitotic index, cytological atypia, and coagulative tumor cell necrosis as the three defining and still most widely used diagnostic parameters (the “Stanford criteria”) [1]. A subsequent clinicopathologic series by Ip et al. refined the histological boundaries between STUMP and leiomyosarcoma in a cohort of 16 uterine cases [2].

A 2024 systematic review of the STUMP literature over the preceding 20 years, drawing on 32 qualifying studies, reaffirmed that STUMP is characteristically a tumor of perimenopausal and postmenopausal women, typically diagnosed between 45 and 55 years of age, with abnormal uterine bleeding, pelvic pain, or an incidentally discovered pelvic mass as the usual presentation [3]. Reported recurrence rates for uterine STUMP range from roughly 7% to over 30% across series, with relapses as either recurrent STUMP or frank leiomyosarcoma sometimes occurring after a disease-free interval of many years, which underlies the recommendation for prolonged surveillance even after apparently complete excision [9].

Extra-uterine STUMP is disproportionately rarer still, and the retroperitoneum is one of the least common reported sites. No formal incidence rate has been established for extra-uterine or retroperitoneal STUMP, given that the published literature consists almost entirely of isolated case reports and small case series rather than population-based data [3]. Won et al. described a retroperitoneal STUMP with pulmonary metastases arising years after hysterectomy in a 62-year-old woman, in whom the disease pursued an indolent, radiologically stable course over at least five years despite chemotherapy [5]. Hariprasad et al. reported a primary retroperitoneal STUMP without any antecedent uterine or gynecologic lesion in a man in his sixties, who remained recurrence-free 24 months after wide local excision; the authors specifically emphasized how few primary retroperitoneal STUMPs, and how few in men, had been described up to that point [4]. More recently, isolated reports of STUMP arising in male patients at other extra-uterine soft-tissue sites have appeared, including an inguinal soft-tissue STUMP in a 74-year-old man that required careful distinction from metastatic lymphadenopathy [7], and a pelvic STUMP of indeterminate origin in an older male patient, for which the authors likewise stressed the exceptional rarity of extra-uterine STUMP in men and recommended prolonged surveillance given the pattern of late recurrence documented in uterine disease [8]. Table 1 summarizes the sex, site, antecedent pathology, and outcome of the retroperitoneal and extra-uterine male STUMP cases identified in the literature alongside the present case.

**Table 1:** Reported cases of extra-uterine and male-patient STUMP relevant to retroperitoneal disease, compared with the present case. STUMP: smooth muscle tumor of uncertain malignant potential; HPF: high-power field.

Study (Year)	Site	Sex / Age	Antecedent lesion	Mitoses /10 HPF	Outcome
Won et al., 2011 [5]	Retroperitoneum (post-hysterectomy)	F / 62 y	Prior uterine leiomyoma, hysterectomy	Not specified	Stable disease with lung metastases over 5 y follow-up
Hariprasad et al., 2023 [4]	Retroperitoneum (primary)	M / 60s	None	Low (<5)	Recurrence-free at 24 months
Ando et al., 2025 [7]	Inguinal soft tissue	M / 74 y	None	Not specified	No recurrence reported
Mourad et al., 2026 [8]	Pelvis	M / older adult	None (origin indeterminate)	Not specified	Surveillance advised; outcome pending
Present case	Retroperitoneum (recurrent, at site of prior excision)	M / 25 y at onset	Histologically confirmed benign leiomyoma, same site, 6 y earlier	18–20	No recurrence at 7 months postoperatively

Set against this background, the present case is notable on at least three counts that, together, have not previously been reported in combination. First, the patient's sex and age place him well outside the typical STUMP demographic: male sex alone accounts for only a small minority of all published STUMP cases, and a man in his mid-twenties is exceptional even within that minority, where reported male cases have generally involved patients in their sixties or older [4,7,8]. Second, unlike the previously reported retroperitoneal STUMPs summarized above, which were either primary lesions with no antecedent pathology or arose after a gynecologic procedure in a woman, this lesion developed at the precise site of a prior excision that had been independently and prospectively diagnosed as a benign leiomyoma, with the original slides re-reviewed and reconfirmed at the time of recurrence. Third, the recurrent lesion showed a striking increase in mitotic activity relative to the original tumor (from a quiescent benign pattern to 18-20 mitoses/10 HPF) without acquiring the cytological atypia or coagulative necrosis that would mandate a diagnosis of leiomyosarcoma. This trajectory, a confirmed benign leiomyoma evolving at the same anatomical site into a mitotically active STUMP over a six-year interval, raises the possibility of genuine biological progression along the leiomyoma-STUMP continuum, a phenomenon that is biologically plausible given the shared smooth-muscle lineage of these tumors but that has, to our knowledge, not been documented before in an extra-uterine, male patient.

The pathological review of both specimens supported the hypothesis of biological progression, as the recurrent tumor was significantly more cellular and mitotically active than the original lesion (compare Figure 2 and Figure 6). Whether this represents true biological progression or reflects tumor heterogeneity and incomplete sampling at the time of the original excision remains uncertain, and this distinction cannot be resolved definitively on morphology alone.

Because retroperitoneal STUMP, and STUMP in male patients generally, lacks a dedicated management algorithm, treatment in this case was extrapolated from the uterine literature and adapted to the anatomical constraints of the retroperitoneum. Complete surgical excision with negative margins remains the cornerstone of treatment; where the tumor abuts bone, as in this case with the right iliac crest, a multidisciplinary approach incorporating periosteal or bone margin resection and reconstructive support (here, polypropylene mesh abdominal wall reconstruction) allows oncologically sound clearance without sacrificing structural integrity. Given the unpredictable behavior of STUMP and its reported risk of recurrence, including recurrence after disease-free intervals of many years, as seen in this patient's own six-year interval between excisions, long-term surveillance is essential and should not be curtailed simply because the index lesion was initially read as benign. In the present case, no recurrence was identified seven months after surgery, but the arc of this patient's disease argues for years, rather than months, of continued follow-up.

## Conclusions

We report a rare case of recurrent retroperitoneal STUMP in a young male arising six years after excision of a benign retroperitoneal leiomyoma at the same site. Retroperitoneal STUMP is uncommon in either sex, and male patients, particularly in the young age group, account for only a small fraction of all reported cases; a man in his twenties with this diagnosis is, to our knowledge, without direct precedent in the published literature. The case demonstrates the diagnostic challenges associated with smooth muscle neoplasms that fall between leiomyoma and leiomyosarcoma, highlights the possibility of biological progression from leiomyoma to STUMP, and underscores that a benign initial diagnosis does not obviate the need for long-term surveillance - a point of particular relevance given how sparse the evidence base is for male and extra-uterine disease. Complete surgical excision with negative margins, supported by a multidisciplinary approach where adjacent bone is involved, together with sustained follow-up, remain crucial for optimal management.
